# Stroop effect in smartphone addiction among college students

**DOI:** 10.1097/MD.0000000000026741

**Published:** 2021-07-30

**Authors:** Ting Zhang, Na Gong, Rui Jia, Huian Li, Xiaoli Ni

**Affiliations:** a School of Humanities and Social Sciences, Xi’an Jiaotong University, Xi’an; b College of Tourism & Landscape Architecture, Guilin University of Technology, Guilin; c Department of Radiology, Ankang People’ Hospital, Ankang; d College Students Mental Health Education Center, Xi’an University of Technologh, Xi’an, China.

**Keywords:** cognition, college students, smartphone addiction, stroop effect

## Abstract

Smartphone addiction (SPA) affects an increasing number of college students, but it remains poorly understood. This study aimed to explore the psychological mechanism of the decrease in cognitive control ability from cognitive psychology in students with SPA.

The smartphone addition tendency scale (MPATS) was used to identify 64 undergraduates with SPA (MPATS > 45) and 64 sex- and age-matched controls (MPATS < 30).

The 2 groups were well matched for age and sex distribution. The RT of the SPA group under the conflict state was 628.2±59.2 ms vs 549.4 ± 44.2 ms under the consistent state (*P* < .005). The RT of the control group under the conflict state was 707.5 ± 66.4 vs 582.0 ± 39.4 ms under the consistent state (*P* < .005). Under the conflict state, errors made by the SPA group were 8.7 ± 5.4, and that of the control group was 6.6 ± 3.7 (*P* < .05). The reaction delay of the SPA group was 25.6 ± 49.2 vs 110.0 ± 41.8 ms (*P* < .05).

Correlation analysis showed a clear positive correlation between SPA and the number of mistakes in the conflict state of the Stroop task.

## 1 Introduction

In the network ecological environment, smartphone surfing has become the daily routine of college students. The 45th “Statistical Report on Internet Development in China” shows that as of March 2020, the number of Internet users in China has reached 904 million, with Chinese teenagers accounting for the most significant number, accounting for 26.9%, among which college students accounted for 19.5%.^[1]^ Smartphones are the primary source of internet access for college students. SPA causes problems such as communication barriers, learning and cognition impairments, interpersonal communication obstacles, fragmentation of social relations, and has become an essential factor affecting the development of the social psychology of the individuals affected by SPA.^[2–5]^

“Smartphone syndrome,” “smartphone dependency,” “smartphone addiction syndrome,” “smartphone anxiety neurosis,” and “smartphone addiction” are all descriptions of the excessive use of smartphones. To a certain extent, the over-reliance on smartphones is consistent with classical “behavioral addictions.”^[6,7]^ Psychiatrists believe that SPA is an “excessive use” behavior, having various adverse effects on physical and mental health as well as the learning progress.^[5,8]^ Psychologists believe that SPA is an “addictive behavior” that causes excitement and pleasure as an extraordinary hobby and habit.^[5,6]^

The Stroop effect was discovered by the American psychologist John Riddley Stroop in 1935.^[9]^ Stroop merged the 2 stimulus dimensions of word and object (color) into a single whole, creating a comic response conflict scenario. Word recognition and font color recognition are 2 different cognitive processes. The information processing rates of these 2 processes differ. Word reading is always faster than color recognition so that the meaning of the words will be dealt with first. When the color of the word is the same as the meaning of the word, recognition of font color will be promoted; otherwise, font color recognition will be impeded. Therefore, the speed of font color recognition differs, and this difference is the Stroop effect.^[10]^ Stroop tasks are used to investigate the cognitive control status of the subjects through the Stroop effect. In cognitive control, color-word Stroop tasks are commonly used to study the neural mechanism of conflict processing.^[11–13]^ The so-called cognitive control is also called executive control. It refers to the process by which individual stores, plans, and manipulates the relevant information from top to bottom according to the objectives of the current task. Cognitive control ability plays an important role when people need to overcome automatic behavior or the current task is unfamiliar, dangerous, or requires planning and decision-making.^[14–17]^

In this study, the Stroop effect was used for the cognitive-behavioral measurement of SPA among college students. This research aimed to explore the psychological mechanism of the cognitive control ability from the perspective of cognitive psychology in college students with SPA. The results might provide a scientific basis for moderate intervention and reasonable treatment.

## 2 Materials and methods

### 2.1 Study design and subjects

This prospective case--control study of students studying at Shaanxi universities was recruited between September 2016 and August 2017. The study was approved by the ethics committee of The First Affiliated Hospital of Xi’an Jiaotong University. All subjects signed the informed consent form.

For the SPA group, the SPA Tendency Scale (MPATS)^[18]^ was used. It uses 5-point scoring for 16 items of 4 dimensions, including abstinence symptoms, prominence behavior, social comfort, and mood alteration. The reliability is 0.834, and the validity coefficient is >0.9. Loads of each factor are 0.51 to 0.79. The cumulative variance contribution rate is 54.3%. Confirmatory factor analysis showed that the 4-factor model in the scale fitted well. The Cronbach α coefficient of the entire scale is 0.83. The Cronbach α coefficients of the 4 factors are between 0.55 and 0.80. The retest reliability of the total scale is 0.91. The retest reliability of the 4 factors is 0.75 to 0.85.^[18–20]^ The questionnaire (Table 1) was filled in a quiet environment. The reliability of the questionnaire was confirmed through phone communication with their parents and by asking classmates in the same dormitory whether the subjects often used smartphones for internet access and whether the roommates were disturbed. The subjects were considered to be SPA when the MPATS score was ≥45.

**Table 1:** Smartphone Addiction Tendency Scale (MPATS) of college students.

1. I tend to check for missed calls or messages immediately after being without my smartphone for a while.
2. I prefer chatting over the smartphone to communicating face to face.
3. While waiting for others, I always make frequent phone calls to check where they are. Otherwise, I feel anxious.
4. I feel uncomfortable if not using a smartphone for a while.
5. I cannot concentrate in class because of phone calls or messages.
6. I feel lonely in the absence of a smartphone.
7. I feel more confident when communicating with others over the phone.
8. I feel uncomfortable after a while without a phone ring and will involuntarily check for missed calls/messages.
9. I often imagine that my phone is ringing/vibrating.
10. I feel more satisfied when there are more phone calls and messages.
11. I am often afraid of the automatic shutdown of my smartphone.
12. A smartphone is a part of me. In its absence, I feel like I have lost something.
13. My classmates and friends often say that I am too dependent on the smartphone.
14. When the smartphone loses internet access or cannot receive signals, I become anxious and irritable.
15. I tend to focus on the smartphone in class, which affects the lectures.
16. I feel more comfortable communicating with others over the smartphone.

For the control group, a total of 64 normal subjects were included. They were matched for sex, age, and education level with the SPA group. The scores of the 64 normal subjects in the MPATS test were <30. During the interview, the subjects used smartphones for internet access <3 times.

### 2.2 Demographic data

The demographic data of all subjects were collected, including sex, age, years of education, hours online every day (hours/day), days online every week (days/week), the score of the MPATS test, and years using the internet (years).

### 2.3 Collection of the behavioral data

**Figure 1:**
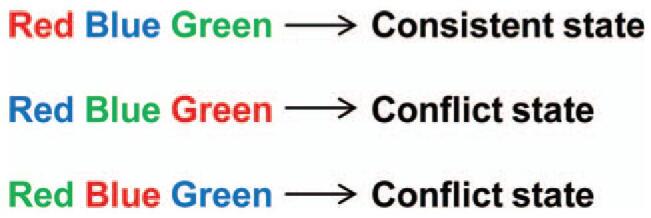
The Stroop word-color task.

The Stroop task in this study included three colors (red, blue, and green). There were 2 states in this task, the consistent state when the word and the color were consistent, and the conflict state when the word and the color were inconsistent (Fig. 1). The E-prime software was used to perform the Stroop task. Two runs were included in this experiment. Four consistent states, 4 inconsistent states, and 9 resting states of blocks were included in each run. There were 7 trials in each task-related block. Each stimulus lasted for 1 second, and the interval between stimuli was 2 seconds. Therefore, blocks related to each task lasted 21 secondse. All blocks under the resting state lasted 17 seconds, except for the first one that lasted 19 seconds. Before each block, there was a “color recognition” instruction for all subjects. All instructions lasted 2 seconds. All runs lasted 367 seconds in total. All subjects were told to press the keys using the index finger, middle finger, and ring finger, respectively. They were required to press keys in response to font color. The order of the keys was red, blue, and green. During the transition, the subjects were required to watch the white cross on the screen. In order to ensure data quality, the subjects performed the behavioral tests in quiet rooms.

### 2.4 Analysis of the behavioral data

The indexes included the reaction time (RT) under conflict state, RT under consistent state, errors under consistent state, errors under conflict state, and reaction delay. The time of conflict state was the RT under conflict state, and the time of consistent state was the RT under consistent state. The Stroop effects of the 2 groups were then calculated. Reaction delay = RT under conflict state - RT under consistent state.

### 2.5 Statistical analysis

SPSS 19.0 (IBM, Armonk, NY) was used for statistical analysis. Continuous variables were presented as means ± standard deviations and analyzed using the Student *t* test. Categorical variables were presented as numbers and percentages and analyzed using the Chi-square test. The Pearson Correlation was used to examine the correlations among two variables. Two-sided *P*-values <.05 were considered statistically significant.

## 3 Results

### 3.1 Characteristics of the subjects

As summarized in Table 2, there was no significant difference in sex, age, and years of education between the 2 groups (all *P* > .05). Indexes including hours on the internet per day, days on the internet per week, MPATS, and years using the internet were all significantly higher in the SPA group than in the control group (all *P* < .005).

**Table 2:** Characteristics of the subjects.

Items (unit)	SPA (n = 64)	Control (n = 64)	*P*
Age, yr	20.3 ± 1.2	19.8 ± 2.2	>.05
Sex (male)	42 (53.8%)	38 (48.7%)	>.05
Years of education, yr	13.3 ± 0.6	14.2 ± 0.3	>.05
Hours on internet per day, h/day	8.6 ± 1.7	2.6 ± 0.4	<.005
Days on internet per week, days/wk	6.2 ± 1.0	2.3 ± 0.7	<.005
Smart phone addition tendency scale (MPATS)	64.4 ± 10.3	26.6 ± 3.2	<.005
Years using internet, yr	6.0 ± 2.7	3.3 ± 1.9	<.005

### 3.2 Behavioral test results

As summarized in Table 3, the RT of the SPA group under the conflict state was 628.2 ± 59.2 vs 549.4 ± 44.2 ms under the consistent state (*P* < .005). The RT of the control group under the conflict state was 707.5 ± 66.4 vs 582.0 ± 39.4 ms under the consistent state (*P* < .005). Under the conflict state, errors made by the SPA group were 8.7 ± 5.4, and that of the control group was 6.6 ± 3.7 (*P* < .05). The reaction delay of the SPA group was 25.6 ± 49.2 ms, and that of the control group was 110.0 ± 41.8 ms (*P* < .05).

**Table 3:** Behavioral test results of the 2 groups.

Items	SPA		Control	
	Consistent state	Conflict state	Consistent state	Conflict state
Reaction time, ms	549.4 ± 44.2	628.2 ± 59.2	582.0 ± 39.4	707.5 ± 66.4
Reaction errors (number)	3.2 ± 2.4	8.7 ± 5.4	3.0 ± 2.0	6.6 ± 3.7
Reaction delay, ms	78.9 ± 45.4	125.6 ± 49.2		

### 3.3 Correlation between the degrees of SPA and Stroop task performance

Correlation analysis showed a clear positive correlation between SPA and the number of mistakes in the conflict state of the Stroop task. The correlation coefficient was 0.549 (*P* < .005) (Table 4).

**Table 4:** Correlation analysis of the degrees of SPA and Stroop task performance.

	SPA	Stroop task
Degrees of SPA Pearson Correlation	1	0.549
*P*		.002
N	64	64
Stroop task Pearson Correlation	0.549^∗^	1
P value	0.002	
N	64	64

## 4 Discussion

SPA is affecting an increasing number of college students,^[2–5]^ but it remains poorly understood. Therefore, this study aimed to explore the psychological mechanism of the decrease in cognitive control ability from cognitive psychology in students with SPA. The results suggest that the cognitive control ability of undergraduates with SPA was impaired.

The undergraduate period is a relatively particular developmental stage, accompanied by multiple changes in psychology, physiology, and social attributes.^[21]^ The relatively immature cognitive control ability during this period may make undergraduate students inadaptable to society and vulnerable.^[21]^ Therefore, the risks for college students to face various affective disorders or addictions significantly increases.^[22]^ Several studies showed that SPA had become a significant problem that seriously affects the development of college students’ or teenagers’ social psychology.^[2–5,18,23–25]^

Yali and Dandan^[26]^ summarized the college students’ reasons for SPA: the specificity of the college students’ development and their personal characteristics, college students’ compliance with group norms, college students’ emotional transfer, and satisfaction of psychological needs. Skierkowski et al^[27]^ suggested that personalities, self-esteem, loneliness, and sense of control greatly impacted SPA. Tang et al^[11]^ found that self-control and vanity were the 2 major subjective factors leading to undergraduate SPA. Hong and Hongli^[28]^ performed tests among college students using the MPATS, loneliness scale, and questionnaire of smartphone use motivation, and showed that loneliness was a vital factor for SPA. Thus, internal factors for college students’ SPA could be summarized as college students’ desire for information, group psychology of college students, college students’ pursuit of fashion, and college students’ emotion transfer.

Liu et al^[16]^ analyzed multiple objective features of smartphones (open, interactive, suppositional, equal, fast, pluralistic, anonymous, instant), the influence of school lifestyle and educational mode, as well as family factors. Skierkowski et al^[27]^ examined social communication according to demographic variables (age, sex, major, grade, and region). Haijun and Ronghui^[29]^ established a linear regression model for analysis and concluded that 4 factors influenced SPA: network social demand factor, traditional social demand factor, self-management demand factor, and entertainment demand factor. Tang et al^[11]^ chose social, environmental factors, and management factors, and showed that society, family, and school significantly impacted undergraduate SPA.

In the present study, behavioral measurement was performed for the cognitive control ability of SPA in college students from the perspective of cognitive psychology. Color-word Stroop tasks were performed for quantitative measurement. The results showed apparent Stroop effects in both the SPA and control groups. Under the conflict state of the Stroop task, the RT of the SPA group was significantly longer than that of the control group, and more errors were made than in the control group. In addition, there was a positive correlation between SPA and the number of errors made under the conflict state of the Stroop tasks. These results strongly suggest that the cognitive control ability of college students with SPA was decreased, and their cognition levels also declined.

This study has limitations. First, the number of subjects was relatively small. In future studies, the sample size needs to be expanded. Second, when subjects were performing behavior tasks, the surrounding environment was not absolutely quiet, which might affect their final behavior. Finally, the dynamic tracking of students with SPA was not performed in this study, so the accuracy of the results was limited in relative time. Those limitations will be addressed in future studies.

In conclusion, using Stroop word-color tasks, the present study strongly suggests that the cognitive control ability of undergraduates with SPA was impaired. From this study, a more comprehensive and in-depth understanding of the impact mechanism of cognitive behavior of SPA was obtained, providing scientific guidance for future intervention and treatment to improve the social development of students with SPA.

## Author contributions

**Conceptualization:** Xiaoli Ni.

**Data curation:** Ting Zhang, Na Gong, Rui Jia, Huian Li.

**Formal analysis:** Ting Zhang, Na Gong, Huian Li, Xiaoli Ni.

**Investigation:** Ting Zhang, Na Gong, Rui Jia, Huian Li.

**Methodology:** Ting Zhang, Na Gong, Rui Jia.

**Project administration:** Xiaoli Ni.

**Writing – original draft:** Ting Zhang, Na Gong, Rui Jia, Huian Li, Xiaoli Ni.

**Writing – review & editing:** Ting Zhang, Xiaoli Ni.
